# Trends in Health Care Worker Intentions to Receive a COVID-19 Vaccine and Reasons for Hesitancy

**DOI:** 10.1001/jamanetworkopen.2021.5344

**Published:** 2021-03-23

**Authors:** Michelle N. Meyer, Tamara Gjorgjieva, Daniel Rosica

**Affiliations:** 1 Center for Translational Bioethics and Health Care Policy, Geisinger Health System, Danville, Pennsylvania; 2 Steele Institute for Health Innovation, Geisinger Health System, Danville, Pennsylvania; 3 Geisinger Commonwealth School of Medicine, Scranton, Pennsylvania

## Abstract

This survey study queried employees of a health care system before COVID-19 vaccine distribution to assess their intentions to receive a vaccine and to understand their reasons for hesitancy to do so.

## Introduction

Although health care workers (HCWs) can serve as ambassadors of COVID-19 vaccine acceptance, surveys have found low acceptance rates among HCWs (eg, 33.5%).^1^ However, those surveys were conducted before the issuance of vaccine emergency use authorizations (EUAs) by the US Food and Drug Administration (FDA). We surveyed all employees of a health care system on the eve of vaccine distribution to encourage them to receive a COVID-19 vaccine, assess their intentions to do so, and understand reasons for hesitancy.

## Methods

This analysis of nonidentifiable administrative survey data did not constitute human participant research and was not subject to institutional review board approval or consent requirements, in accordance with 45 CFR §46.102(e)(1). This study follows the American Association for Public Opinion Research (AAPOR) reporting guideline.

On December 4, 2020, an announcement concerning anticipated vaccine availability was emailed to all employees. The announcement contained a link to a 5-question online survey and stated that employees’ time-sensitive response was needed to guide vaccine distribution. Reminders were included in 2 emailed employee newsletters.

Analyses were conducted using R statistical software version 4.0.2 (R Project for Statistical Computing). We set statistical significance at α = .005 for 2-tailed *t* tests.

## Results

A total of 16 292 employees (68.5% response rate) completed the survey. The employee population is 73% female (17 362 employees) and 89% White (21 168 employees), with a mean age of 43 years. When asked whether they would “decide to receive the COVID-19 vaccine when one is available to [them],” 55.3% of respondents (9015 employees) said yes, 16.3% (2658 employees) said no, and 28.4% (4619 employees) were undecided. Patient-facing employees (58.2% of respondents [9485 employees]) were more likely than employees who do not interact with patients to say yes (57.3% [5432 employees] vs 51.4% [3132 employees]; difference, 5.9%; 95% CI, 4.2%-7.4%; *P* < .001); however, they were also more likely to say no, although the difference was not significant (17.3% [1639 employees] vs 15.6% [948 employees]; difference, 1.7%; 95% CI, 0.5%-2.9%; *P* = .006). Intention varied little by campus, and we found no consistent patterns of intention among different patient-facing areas of work.

**Figure 1.**
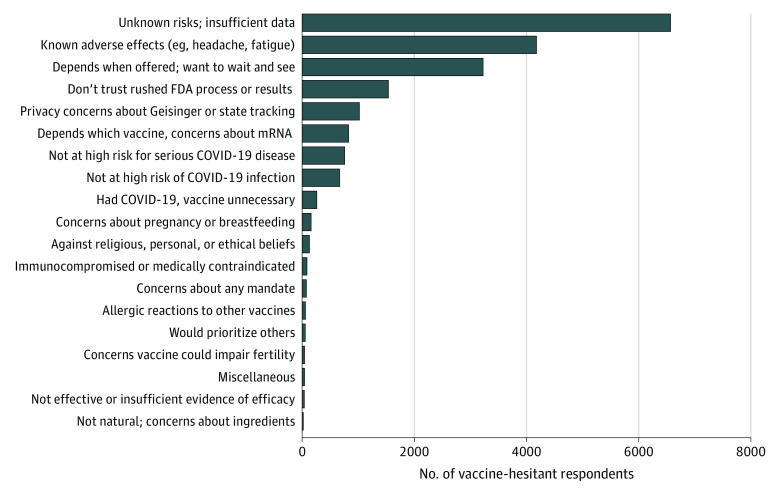
Reasons for COVID-19 Vaccine Hesitancy Sixteen percent of hesitant respondents provided additional reasons beyond those provided in the survey instrument. Those responses were categorized according to a developed codebook into 10 additional reasons, which are shown as the bottom 10 entries in the graph. FDA indicates US Food and Drug Administration.

Most (90.3% [6569 employees]) of those who responded no or undecided reported concerns about unknown risks of the vaccines, 44.3% (3226 employees) reported they wanted to wait until others’ vaccine experiences are known, and 21.1% (1539 employees) reported that they do not trust the rushed FDA process. More than one-half (57.4% [4187 employees]) cited concerns about known adverse effects, such as headache and fatigue (Figure 1).

**Figure 2.**
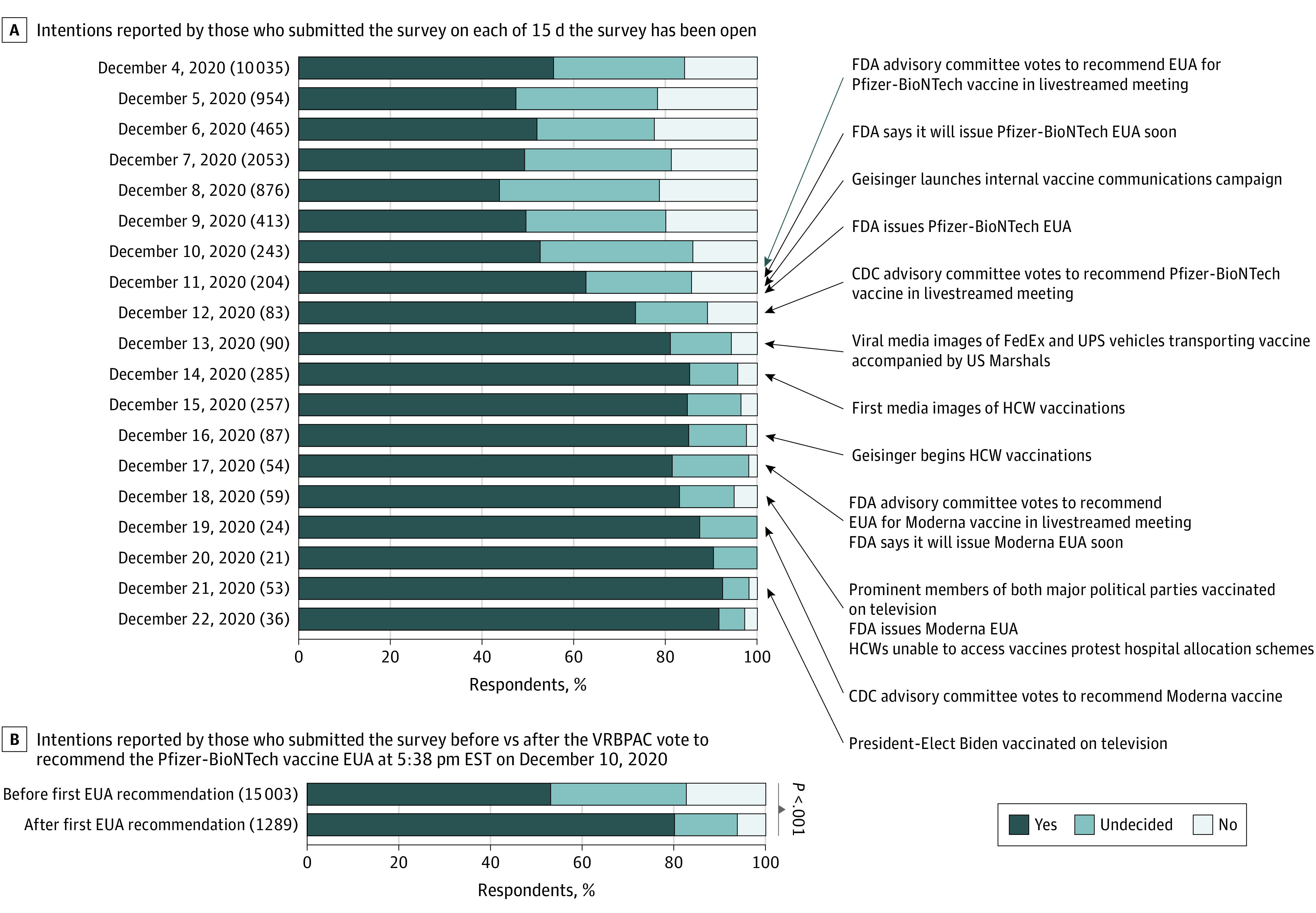
Health Care Workers’ (HCWs) Intention to Take a COVID-19 Vaccine, Over Time Panel A shows intentions reported by those who submitted the survey on each of 15 days the survey was open. Numbers in parentheses next to each date refer to the number of respondents who completed the survey during the relevant period. Panel B shows the intentions reported by those who submitted the survey before vs after the US Food and Drug Administration (FDA) Vaccines and Related Biological Products Advisory Committee (VRBPAC) vote to recommend the Pfizer-BioNTech vaccine emergency use authorization (EUA) at 5:38 pm EST on December 10, 2020. CDC indicates Centers for Disease Control and Prevention.

We observed a steady increase in intention to receive a vaccine during the period of data collection coinciding with several high-profile, vaccine-related events (Figure 2A). The first was the livestreamed vote on December 10, 2020, by an independent FDA advisory committee to recommend the first COVID-19 vaccine EUA. Among 15 003 employees who completed the survey before that time, 53.2% (7981 employees) reported they would receive a COVID-19 vaccine; by contrast, 80.2% (1034 employees) of the 1289 respondents who subsequently completed the survey reported such an intention (difference, 27.0%; 95% CI, 24.7%-29.3%; *P* < .001) (Figure 2B). Among the 1289 employees who responded after December 10, patient-facing employees again (1054 employees) were more likely than non–patient-facing employees (192 employees) to intend to receive a vaccine (82.3% vs 69.8%; difference, 12.5%; 95% CI, 5.6%-19.4%; *P* < .001). As of February 18, 2021, 67.2% of employees (15 983 employees) have received at least 1 COVID-19 vaccine dose.

## Discussion

The substantially higher rate of COVID-19 vaccine acceptance observed here compared with earlier surveys of HCWs regarding hypothetical vaccines may reflect the different timing and framing of this survey. It was administered for purposes of making imminent decisions about vaccine distribution, was attributed to the Division Chief of infectious diseases, and emphasized the scarcity and high efficacy of specific COVID-19 vaccines.^2,3^

The observed substantial increase in acceptance during the survey period should be interpreted cautiously because of the reduced sample size over time and because survey latecomers may not be representative. Still, intervening events may have signaled that receiving a vaccine is safe, normative,^4^ historic, and an indication of HCWs’ important role in the pandemic response. The trend of increased intention to receive a vaccine as the EUA processes unfolded and the greater number of employees who actually received a vaccine compared with respondents who intended to do so suggest that the highly visible nature of the actual processes may have reassured many respondents.^5,6^
